# A global dataset of immigration flow composition by age, sex and educational attainment

**DOI:** 10.1038/s41597-026-07969-8

**Published:** 2026-09-07

**Authors:** Dilek Yildiz, Guy J. Abel

**Affiliations:** 1 International Institute for Applied Systems Analysis, Wittgenstein Centre for Demography and Global Human Capital (IIASA, OeAW, University of Vienna), Laxenburg, Austria; 2 Department of Sociology and Research Hub of Population Studies, University of Hong Kong, Hong Kong, Hong Kong, Special Administrative Region of China

## Abstract

People at different ages and with different educational background have different migration patterns. However, international migration flows by age and educational attainment are scarce and not always comparable. We use a super learning algorithm with multiple initial models to estimate age and education composition of male and female immigrants for 199 countries over six five-year periods from 1990–1995 to 2015–2020. We access the performance of our approach through cross-validation and comparing initial learners with the super learner algorithm using multiple evaluation metrics. We also compare our age composition estimates against equivalent measures from Eurostat. The estimates indicate that while the proportion of immigration flows with higher education increase in all regions, Europe has the highest immigration flow with secondary or higher education.

## Background & Summary

As populations pass through their demographic transitions, fertility and mortality levels decline to low levels and (natural) population growth stabilises. Consequently, international migration becomes the key component of population change. Migration, as with other components of population change, varies widely by the age, sex, and education of individuals^1^. To better understand the impact of migration on origin and destination countries and to provide effective population projections, measuring migration flows by these three key demographic features is required. However, data on international migration flows by age, sex, and education are sparse.

International migration flow data by age and sex are more readily available than education-specific measures. The Organisation for Economic Co-operation and Development (OECD)^2^ provides total and sex-specific immigration and emigration flows of foreign population by nationality for its member countries. The International Labour Organisation (ILO) compiles immigration and emigration flows of the working-age population by sex and additional characteristics, such as country of birth, and economic activity from various sources including population censuses, administrative records and surveys. Immigration flows for 32 countries and emigration flows for 13 countries are available by educational attainment, beginning from 1999^3,4^. Eurostat provides emigration data by age and sex for 44 countries and immigration data by age and sex for 36 countries since 1990, with substantial missing data for early years and non-EU countries^5,6^. The Determinants of International Migration (DEMIG) project collected data on male and female immigration and emigration flows for 34 countries from the 1940s to 2018^7^. Some countries collect information on immigration flows using population and housing censuses, usually every ten years. Different censuses measure migration flows using different migration intervals (duration since arrival), and when available, characteristics of migrants are often only reported in broad, non-comparable categories. For example, the United Nation’s Latin American and Caribbean Demographic Centre (UN CELADE) provides immigration flow data from censuses by age and sex for 19 countries but does not include any information on returning nationals^8^. Overall, international migration flow data by age and sex tend to be predominantly available in high-income countries with robust statistical infrastructures that can continuously enumerate the number of migrants arriving and departing.

Published education-specific measures of international migration flows do not exist in almost all countries. Consequently, the majority of research on education-specific international migration relies upon migrant stock measures^9–12^, which are easier to collect than migration flows but disguise the dynamic changes in size and scale of period-specific migration patterns. Migrant stock measures by education typically coarsely divide educational attainment into two categories, with or without higher education, which prohibits any detailed analysis of the changing structures of human capital in origin and destination countries. Without more detailed, education-specific migration flow data, our ability to effectively explain and predict human capital gains and losses, as well as their associated costs and benefits, is severely restricted.

To provide a more detailed picture of international migration flows, indirect estimation methods have been proposed. In Europe, Bayesian hierarchical models have been developed to harmonise across the different definitions and collection systems in the reported migration flow data collated by Eurostat, to estimate annual bilateral migration flows between 2002 and 2008; and between 2009 and 2019 by age and sex^13–19^. Similar models have been adapted to estimate annual migration flows in Latin America and between South America and Europe based on data from CELADE and Eurostat^20,21^. These modelling frameworks have also been adapted to estimate annual migration between Asian countries and Pacific nations^22^. They are based on earlier, more ad-hoc approaches to estimating European migration flows. At the global level, estimation methods have been proposed to link together in a demographic accounting framework bilateral migrant stock data provided at five-year intervals with measures on population, births and deaths in each country^23–27^. The resulting global flow estimates quantify the number of migrants required to move between each country during the five-year intervals to match the changes in the migrant stock, total population, births, and deaths data published by the United Nations Department of Economic and Social Affairs (UNDESA). Some of these estimation methods use a demographic accounting approach that enables the estimated flows to sum to corresponding country-specific net migration totals published by UNDESA. The demographic accounting frameworks have been extended to produce sex-specific bilateral migration flow estimates between all countries, based on changes over five-year periods between sex-specific data on global migrant stocks and population totals, while controlling for births and deaths during the time interval^24,28^.

There are currently no published data or estimates of international migration flows with age- or education-specific disaggregation at the global level. The lack of available data hinders our ability to accurately project future migration patterns and population changes using cohort-component models, which require, at a minimum, assumptions about the age- and sex-specific compositions of future migration rates. A prominent example is the UNDESA World Population Prospects^29^, which uses a net migration measure in its projection models. However, UNDESA only publicly provide the total net migration for past and future levels of all countries (and corresponding rates), without disclosing the age- and sex-specific values used in their population projection models. Other bodies, such as the US Census Bureau^30^ and the Institute for Health Metrics and Evaluation^31^, that produce global population estimates and projections also use a net migration measure and do not provide any age- or sex-specific disaggregation. The global human capital population projections conducted by the Wittgenstein Centre (WIC)^32,33^ utilise sex-specific immigration and emigration rates, rather than a net migration measure, enabling a more nuanced specification of future migration scenarios and the straightforward incorporation of age-specific patterns. Base data for the projections are built on sex-specific immigration and emigration totals obtained from estimated flows resulting from changes in global bilateral migrant stock data, as described above. Age and educational compositions for base data and future assumptions have previously been based on applying standard migration age schedules and educational shares corresponding to the populations of the receiving countries. These simple assumptions have scant empirical support. In this paper, we present a new approach to estimating the age and educational compositions of sex-specific migration flows, drawing on available data from the Integrated Public Use Microdata Series (IPUMS) International database^34^ census samples. Our empirical-based estimates allow, for the first time, a better understanding of the demographic composition of international migration flows related to human capital losses and gains. They also potentially serve as a more reliable base for the demographic composition of international migration flows used in global population projections by age, sex, and education.

Recent research on human migration is increasingly using machine learning (ML) approaches to improve analysis of the drivers and patterns of migration in different contexts. Studies have applied ML to model internal migration^35,36^, to predict asylum and irregular migration flows including migrants’ legal status^37–39^, and to assess the importance of climatic and environmental factors on migration^39–42^. Random forests (RF) are frequently employed to handle large, multidimensional datasets, both to predict migration flows and to examine variable importance. In addition, other advanced ML approaches such as deep-learning gravity models^43^ or RF combined with demographic modelling such as survival models^36^ have expanded the methodological framework.

We focus on estimating the demographic compositions of immigration flows, i.e., the age and education composition of flows by sex, rather than the levels of migration flows by the same demographic properties. We take this approach for three reasons. First, as noted previously, global estimates of migration flows by sex have already been developed; therefore, our computational estimates can be directly applied to obtain immigration levels by age, sex, and education. Second, the data on which we base our estimation defines migration using the spread of timing intervals, from census questions that identify whether respondents were abroad at a fixed interval, such as one or five years before the census data collection period. The different timing intervals make a direct comparison of migration levels challenging due to the *one-year-five-year migration problem*^44–46^. Comparisons between the compositions, rather than the levels of flows, based on migration data derived from different timing intervals are far more straightforward. Third, the age composition of migration flows typically follows a distinct life-course trend. Young adults, particularly those in their late teens and twenties, exhibit the highest migration rates as they seek education and job opportunities. Migration then declines in middle age. In some exceptional cases, migration flows may rise again among retirees seeking lifestyle changes or better living conditions. These regularities are much better studied and modelled by focusing on the age compositions of migration rather than the levels of migration that might be skewed by different exposure populations to immigrate across various age groups.

In the remainder of this article, we outline our method for estimating the age and education compositions of sex-specific immigration flows. We illustrate our results and compare them with reported migration flow statistics in countries where comparable data are available. We then summarise the results of our validation exercises and offer some guidance to potential users of the database. Finally, we discuss the limitations of our estimates.

## Methods

We estimate the age and education composition of immigration flows in two steps. First, we apply Roger-Castro migration age schedules to the immigration compositions derived from the IPUMS International database^34^ to account for data limitations due to small sample sizes. Then, we use these estimates as inputs to a super learning (SL) algorithm, which utilises multiple base random forest models. Finally, we predict the age and education compositions of male and female immigration flows in five-year periods between 1990–1995 and 2015–2020 for 199 countries.

In this section, we first discuss the input data on which we build our modelling framework before detailing the two steps of our estimation procedure.

### Migration data

**Fig. 1:**
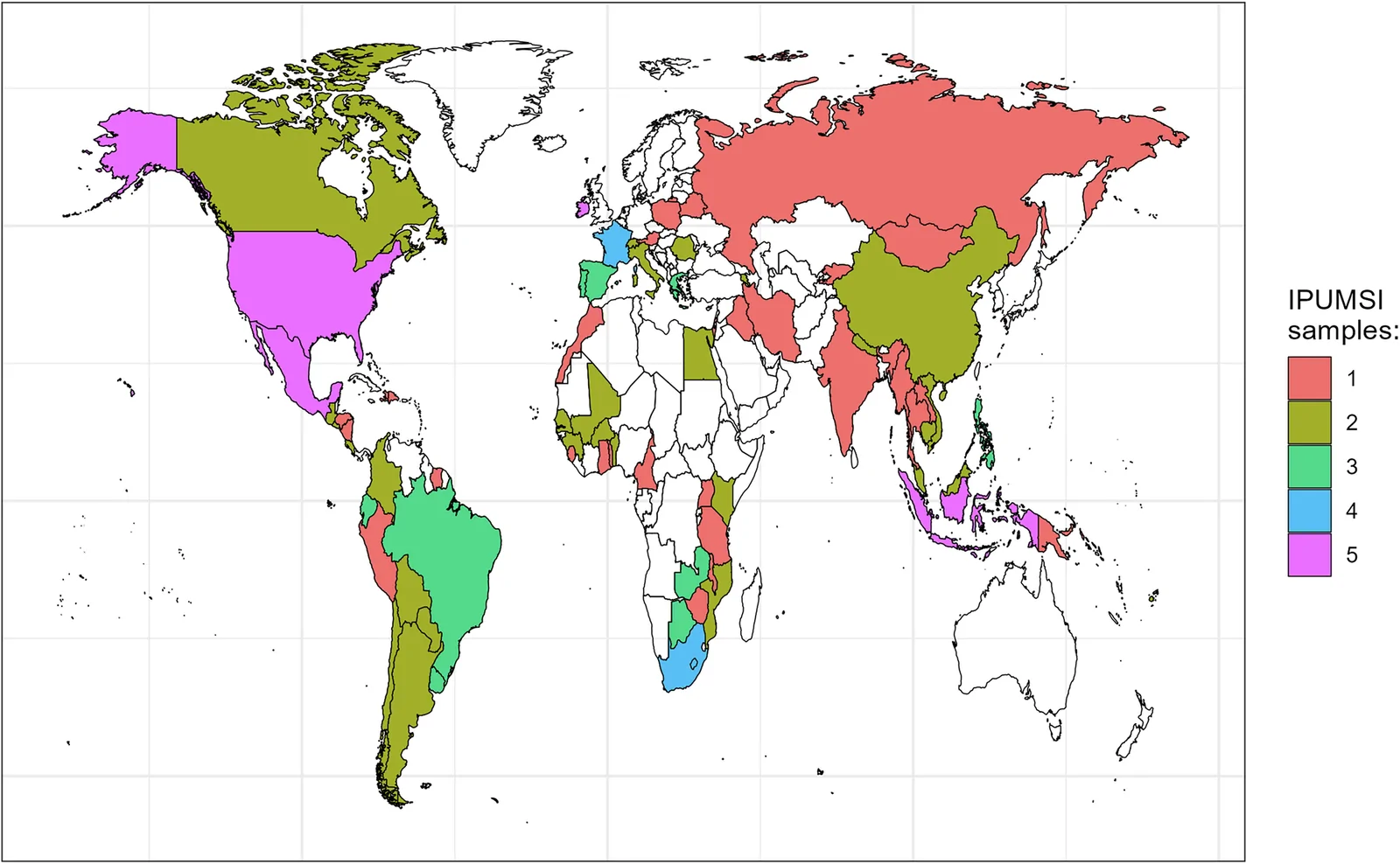
Number of census samples used for each country.

**Fig. 2:**
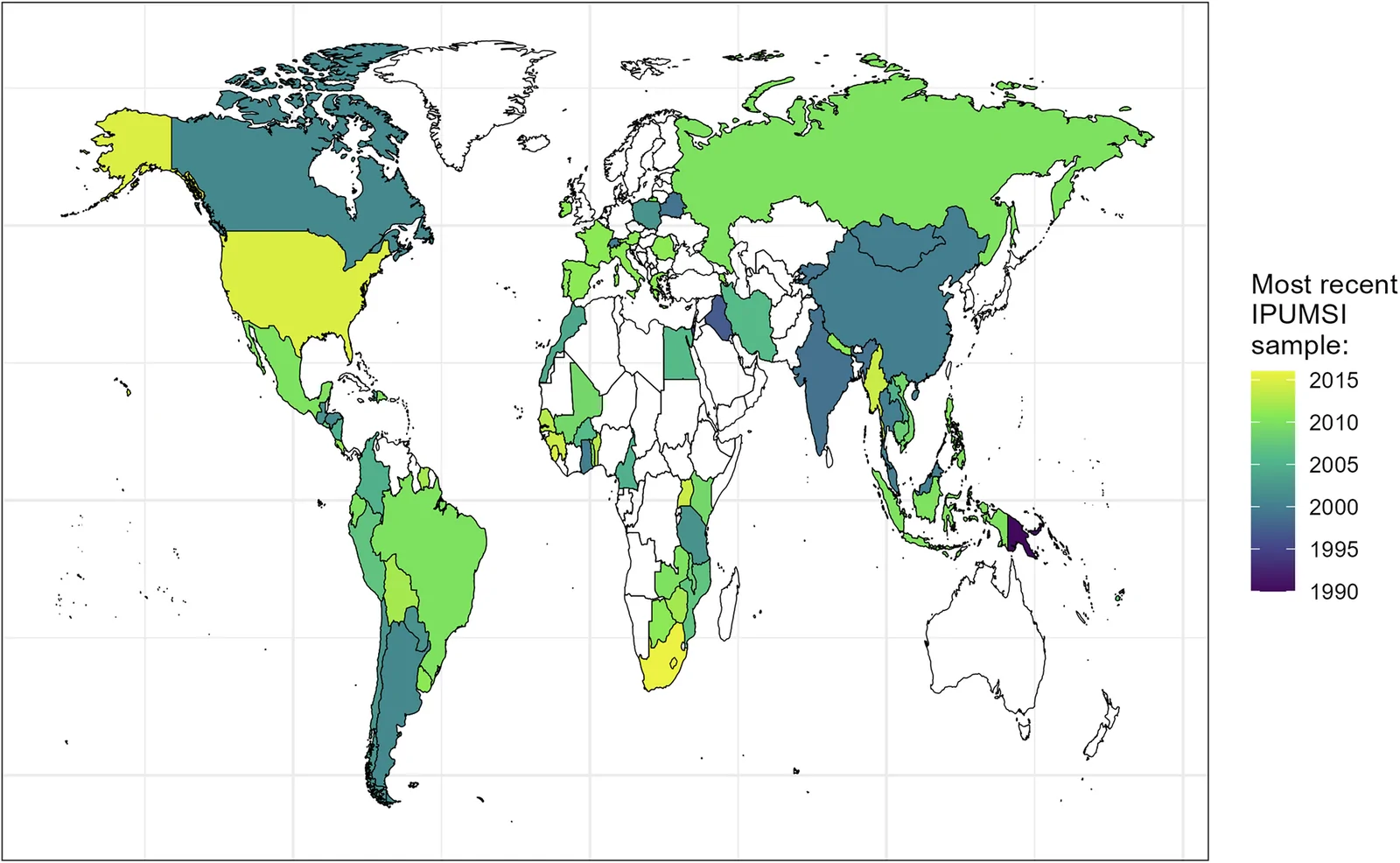
The most recent census sample for each country.

To predict the age and educational composition of immigration flows, we leverage census samples from the IPUMS International database. Samples in IPUMS International typically cover close to 10 per cent of the entire census population; see Supplementary Table 1 for the sampling fractions of the IPUMS International samples used in our study. We selected 143 census samples from 74 countries between 1990 and 2016. Figure 1 presents a map displaying the number of census samples available for each country and Fig. 2 shows the most recent year in which a census sample is available. The census samples have a strong representation across Latin America, North America, sub-Saharan Africa, South and Southeast Asia while some gaps remain in Central Africa, Europe and Middle East. In terms of timing, all five-year periods are well represented except the last period 2015–2020. Migration-related variable(s) in census samples allowed us to identify whether the respondent had recently arrived in the country at the time of the census. In addition, harmonised variables on age, sex and education characteristics were also available in all samples, which allowed us to cross-tabulate the number of recent immigrants by their basic demographic characteristics. Education level was based on the IPUMS International variable ‘edattain”, for the person’s educational attainment in terms of the level of schooling completed, with four possible levels (less than primary completed, primary completed, secondary completed and university completed).

The identification of recent immigrants was derived from one of two approaches, depending on the form of migration question used in the census. In the first approach, we utilised an IPUMS International variable indicating an individual’s residence at a fixed point in time before the census, such as one or five years ago. If the previous region of “Abroad” was given, we coded the respondent as an international immigrant. If a variable was unavailable at a fixed time before the census, we used a combination of two variables: a variable indicating whether the respondent’s previous residence was abroad (or within the same region), and a second variable with the number of years the respondent had resided in their current location. If the respondent had changed their location within five years, and the previous residence was Abroad, we coded the respondent as a recent immigrant. In total we identified 2.53 million individual recent immigrants, which, when weighted to the population level represented 40.0 million migrants, drawn from 463 million person records.

Due to a small number of observations, migrants younger than 15 and older than 75 years are initially collected in broad age groups, Under 15 and 75+. The Under 15 age group is further divided into three five-year age groups using Roger-Castro migration age schedules, and these age groups are incorporated into our model. Above the age of 15, four education categories are considered: no education, primary education (including primary education completed), secondary education (including secondary education completed), and post-secondary education. Following other demographic models of past and future age and education patterns, we include “Under 15” as an additional educational category representing individuals in the first three age groups^47^. Post-secondary education is only available to individuals starting from the 20–24 age group. The age and education proportions were calculated by dividing the size of each age-sex-education immigration flow by the total sex-specific immigration flow. Hence, the two sets of sex-specific proportions sum up to one for each country-year set of observations. Eight censuses (CUB2002, CUB2012, GBR2001, GBR2011, KHM2004, KHM2013, ROU1992, THA1990) with fewer than 100 observed immigrants or with coverage of less than 75% of the age- and education-specific proportions were excluded from the dataset. Out of the eight censuses excluded only GBR2001 and GBR 2011 censuses had more than 100 observations. In total there are 52 potential observations based on 12 age groups (from 20–24 age to 75+) and four educational attainment categories, in addition to the three educational attainment categories in the 15–19 age group and the Under 15 age category.

### Roger Castro migration age schedules

Age-specific migration data is often coarse due to issues of age heaping and limitations in data collection systems in effectively capturing migration of small sub-groups of a relatively rare event. To provide predictions of age-specific compositions of immigration flows and account for the missing data, we utilised a Bayesian implementation of the Rogers-Castro migration age schedule^48,49^. The Rogers-Castro migration age schedule offers a parametric model for smooth migration age patterns, transitioning to regular patterns with a peak in early adulthood, followed by a gradual decline thereafter.

Here the Rogers-Castro migration age schedules are used as a preprocessing step to smooth age-specific migration patterns especially for small sample groups. Although, the SL algorithm can be used directly with unprocessed input data, we found that the Rogers-Castro migration age schedules improve predictive performance. We present the cross-validation results based on the unprocessed data compared to our main approach in Table 3.

We employed the seven-parameter Roger-Castro migration age schedule (Eq. 1), which includes pre-working and working age components, as more complex migration schedules, such as those incorporating retirement peaks, were not suitable for any of the country-years in our study. For ages below 15, the input migration data are available only as a single broad 0–14 age group. Therefore, we used the migration rate calculated for the 0–14 age group as the initial value for the first three age groups 0–4, 5–9 and 10–14 in the Bayesian implementation of the Rogers-Castro model migration age schedules. The Rogers-Castro model produces migration rates for age group- (0–4, 5–9, …, 70–74, 75+), sex-, education- and country-specific migration rates. The estimated rates from the Rogers-Castro model were then converted into proportions and used as inputs in the super learning algorithm.

1 $$m(x)={a}_{1}\,\exp ({\alpha }_{1}x)+{a}_{2}\,\exp (-{\alpha }_{2}(x-{\mu }_{2})-\exp (-{\lambda }_{2}(x-{\mu }_{2})))+c$$

$m\left(x\right)$ represents the migration rate by age group, sex and education level for a specific country and time period, ${a}_{1}$ and ${a}_{2}$ represent the peaks of migration rates, ${\alpha }_{1}$, ${\alpha }_{2}$ and ${\lambda }_{2}$ represent the shape of the components (rate of change), ${\mu }_{2}$ is the peak at labour force and c is the baseline level of migration.

### Super learning and random forest

Super learning, also known as stacked generalisation, is an ensemble machine learning approach that combines multiple machine learning models (also interchangeably referred to as algorithms and learners) or the same model with different specialisations^50,51^. Super learning utilises a k-fold cross-validation to generate predictions from candidate (base) learners, which are then used as inputs to a meta-learner model that combines their predictions. The final predictions are the weighted combination of predictions (coefficients of the meta-learner) from the selected base learners. It has been demonstrated multiple times that super learner algorithms outperformed individual base models^52–54^.

In this article, we combine multiple RF algorithms as base learners with different hyperparameters to create an optimised predictive model. The RF algorithm is a widely used ensemble machine learning method that aims to reduce variance while minimising bias, thereby enhancing prediction accuracy and reducing overfitting^55^. During the training phase, the RF approach constructs multiple decision trees using random subsets of data during training and averages their outputs to make multiple predictions. These trees are later used to make predictions on new data. Its robustness to missing data and ability to handle complex and non-linear relationships between variables make it particularly well suited for migration research.

The RF learners were chosen due to their superior performance, as determined by investigating a set of mixed learners. The SL model using only RF learners outperformed the diverse SL with mixed learners, as indicated by consistently lower prediction error across all evaluated metrics. Table 1 reports the learners included in two SL libraries: the main model which is an RF-only library and a diverse library that includes gradient boosting, Multivariate Adaptive Regression Splines (MARS), penalised regression and Bayesian Generalised Linear Model in addition to RF learners.

**Table 1:** Learners in the main SL model and in Diverse SL model.

Panel A: RF algorithms considered in both the main SL and the Diverse SL libraries					
Learner	Specification	MSE (CV Risk)	SE	Main SL coef.	Diverse SL coef.
Ranger autotune	ExtraTrees, mtry = 31, min.node.size = 5, selected by 3-fold CV over mtry ∈ {2, 16, 31, 46, 61} × splitrule ∈ {variance, extratrees}	0,069	0,001	0,951	0,929
Ranger 5	RF, 500 trees, mtry = 5	0,125	0,002	0,000	0,000
Ranger 10	RF, 500 trees, mtry = 10	0,083	0,001	0,000	0,000
Ranger 15	RF, 500 trees, mtry = 15	0,077	0,001	0,000	0,000
Ranger 20	RF, 500 trees, mtry = 20	0,076	0,001	0,000	0,000
Ranger 25	RF, 500 trees, mtry = 25	0,077	0,001	0,000	0,000
Ranger 30	RF, 500 trees, mtry = 30	0,078	0,001	0,000	0,000
Ranger 35	RF, 500 trees, mtry = 35	0,079	0,001	0,000	0,000
Ranger 40	RF, 500 trees, mtry = 40	0,080	0,002	0,000	0,000
Ranger 45	RF, 500 trees, mtry = 45	0.081	0.002	0.049	0.071
Ranger nt1000	RF, 1000 trees, mtry = 20	0,098	0,002	0,000	0,000
Ranger mn10	RF, 500 trees, mtry = 20, min.node.size = 10	0,108	0,002	0,000	0,000
**Panel B: Additional learners only in Diverse SL library**					
Learner	Specification	MSE (CV Risk)	SE	Main SL coef.	Diverse coef.
XGB 3	XGBoost, max_depth = 3, nrounds = 100, eta = 0.1	0,211	0,003	NA	0,000
XGB 5	XGBoost, max_depth = 5, nrounds = 200, eta = 0.05	0,137	0,002	NA	0,000
XGB 6	XGBoost, max_depth = 6, nrounds = 500, eta = 0.01	0,144	0,002	NA	0,000
Earth 1	MARS, main effects only	0,237	0,003	NA	0,000
Earth 2	MARS, two-way interactions	0,237	0,003	NA	0,000
Lasso	Penalised regression, LASSO (α = 1), λ by 10-fold CV	0,413	0,005	NA	0,000
Ridge	Penalised regression, ridge (α = 0), λ by 10-fold CV	0,413	0,005	NA	0,000
Elastic net	Penalised regression, elastic net (α = 0.5), λ by 10-fold CV	0,418	0,005	NA	0,000
BayesGLM	Bayesian GLM, Cauchy priors	0,413	0,005	NA	0,000
GBM	GBM, 500 trees, interaction depth = 3, shrinkage = 0.001	0,726	0,007	NA	0,000

As base learners, in the main SL model, we include twelve RF algorithms with different mtry parameters, splitting rules as well as number of trees and node sizes (Table 1). Initial RF learners with different specialisations were trained and validated using ten-fold cross-validation. Nine learners use the standard RF specification (500 trees, minimum node size = 1) with mtry values varying between 5 and 45. In addition to these nine base learners, we also include one autotuned RF base learner selected by 3-fold cross-validation over a grid of mtry and split rule combinations (variance and ExtraTrees) using the Lrnr_caret function, one RF learner with 1000 trees instead of default 500 trees, and one RF learner with a minimum node size set to 10.

The mtry parameters in RF algorithms define the number of variables (m) that the algorithm tries to find the best candidate for splitting the nodes of the model. In other words, it controls how much information each tree can access at a given decision point and they have been found to have the most significant influence on predictive performance^52,53^. The higher the mtry value, the more chances that a tree can find a more useful variable, while low mtry values may create less correlated trees. The default variance split rule searches for the splits that minimize the variance of the target variable within the nodes, while ExtraTrees^56^ rule draws thresholds randomly and selects the best variable among random cuts, increasing the randomization for less correlated trees. Minimum node size controls  the minimum number of observations required for a node to be split further with large numbers preventing further splitting of small nodes and reducing overfitting.

In the SL model algorithm, 41 variables (61 when dummy variables are considered) listed in Table 2 are treated as candidate predictors for the RF base learners. For each split within a tree, the RF algorithm considers a random subset of predictors from this list, as determined by the mtry parameter. The maximum mtry considered in base RF learners is 45, meaning that at most 45 predictors are considered at any given split. As shown in Table 1, only the autotuned RF learner with mtry value of 31 and the Ranger 45 model are selected in both SL libraries with the meta learner coefficients higher than 0 (shown in main SL and Diverse SL coef. columns). Autotuned RF received coefficients of 0.951 in the main model and 0.929 in diverse SL while Ranger 45 received coefficients of 0.049 and 0.071. The SL places no emphasis on any other learners. The autotuned RF model considers 31 predictors out of 61 predictors (including dummy variables).

**Table 2:** Predictor variables and the data sources used in the SL model.

Predictor variables and variable labels used in datasets and figures	Source
Period (year5)	IPUMS
Sex (sex)	IPUMS
Age group (age)	IPUMS
Education (education)	IPUMS
Migration interval (mig_int)	IPUMS
Population size by age group, education and sex (pop)	WIC
Population by previous age group, education and sex (pop_past)	WIC
Population by next age group, education and sex (pop_next)	WIC
Proportion of the population by age group, education and sex (prop)	WIC
Proportion of the population by previous age group, education and sex (prop_past)	WIC
Proportion of the population by next age group, education and sex (prop_next)	WIC
Total population (popt)	WIC
Immigrant stock (imm_stock)	UN
Size of the emigrant population (emi_stock)	UN
GINI (gini)	Gap Minder
Sex ratio (sexratio)	UN WPP
Sex ratio at birth (srb)	UN WPP
Life expectancy at birth by sex (e0)	UN WPP
Life expectancy at age 80 by sex (e80)	UN WPP
Population density (pop_density)	UN WPP
Median age (median_age)	UN WPP
Rate of natural change (rate_of_natural_change)	UN WPP
Population growth rate (pop_growth_rate)	UN WPP
Births to 15–19 year old mothers (births1519)	UN WPP
Crude birth rate (cbr)	UN WPP
Total fertility rate (tfr)	UN WPP
Mean age at childbearing (mac)	UN WPP
Crude death rate (cdr)	UN WPP
Infant mortality rate (imr)	UN WPP
Under-five mortality rate (u5mr)	UN WPP
Net number of migrants (net_migrants)	UN WPP
Net migration rate (nmr)	UN WPP
Human Development Index (hdi)	UNDP
Gross National Income per Capita (gnipc)	UNDP
Adolescent birth rate (abr)	UNDP
Maternal mortality ratio (mmr)	UNDP
Carbon dioxide emissions per capita production (co2_prod)	UNDP
Expected years of schooling (eys)	UNDP
Percentage of population with at least some secondary education by sex (se)	UNDP
Mean years of schooling by sex (mys)	UNDP
World region (region)	IPUMS

In the SL algorithm, the response variable for all base learners is the log of the proportion of immigrants in each age and education group within the total migrants in a country, categorised by five-year periods and separately for each gender. We used the log of the proportion of immigrants rather than the proportions themselves to address the skewness caused by the low proportion of immigrants in certain age and education groups. After removing abovementioned eight censuses, each country-period-sex combination had on average 5.5 cells (median = 3) with zero proportions. These were dealt with by adding a small error term (0.001) to the proportions prior to logarithmic transformation to avoid undefined values in the log scale. The sensitivity to this choice is partially addressed by benchmarking SL algorithm to more sophisticated distributional models as described below.

We compared the SL model algorithm against three alternative count-data distributional models. Specifically, Zero-Inflated Poisson (ZIP), Zero-Inflated Negative Binomial (ZINB) and Negative Binomial (NB) models were fitted across multiple covariate specifications (full, reduced, minimal and year + age only) and pseudo-count scaling factors (1,000 and 100,000). ZIP and ZINB models failed to converge in all covariate specifications except the models using only year and age covariates. The converged ZIP and ZINB models had Mean Square Error (MSE) around 15 times higher than the main SL algorithm (SL = 1.95e-5 vs ZIP = 3e-4, ZINB = 3.37e-4). Similarly, the best performing NB model had also higher MSE (0.02) than the SL algorithm. Evaluation metrics were also calculated for subsets for high proportion cells in which proportions are equal or higher than 0.10. Also for this subset, SL achieved lower errors than the best performing alternatives in MSE, Root Mean Square Error (RMSE), and Mean Absolute Error (MAE).

The last age group considered is an open-ended age group 75 years and older. The continuous predictor variables were standardised to have a mean of 0 and a standard deviation of 1, and the categorical predictor variables were one-hot encoded (the levels of categorical predictors were converted to a set of binary indicators without dropping any levels increasing the total number of variables to 61). The predictors are listed in Table 2. The SL was trained using 10-fold cross-validation. The default meta learner, non-negative least squares (NNLS) regression (Lrnr_nnls), for continuous outcomes is used to optimise the weighting of base RF learner predictions. The meta learner coefficients for all base learners are presented in Table 1. Model performance was evaluated using a cross validation prediction check (CV risk) across all folds. Table 3 shows that the SL outperformed all initial RF base learners.

**Table 3:** Cross-validated predictive performance (CV risk).

	Main model (SL with RC)	SL without RC
Learner	MSE (CV Risk)	MSE (CV Risk)
Ranger autotune	0,069	0,184
Ranger 5	0,125	0,245
Ranger 10	0,083	0,194
Ranger 15	0,077	0,185
Ranger 20	0,076	0,183
Ranger 25	0,077	0,183
Ranger 30	0,078	0,184
Ranger 35	0,079	0,184
Ranger 40	0,080	0,185
Ranger 45	0,081	0,186
Ranger nt1000	0,098	0,211
Ranger mn10	0,108	0,223
SuperLearner	0,021	0,050

## Age and education proportion of immigration flows

**Fig. 3:**
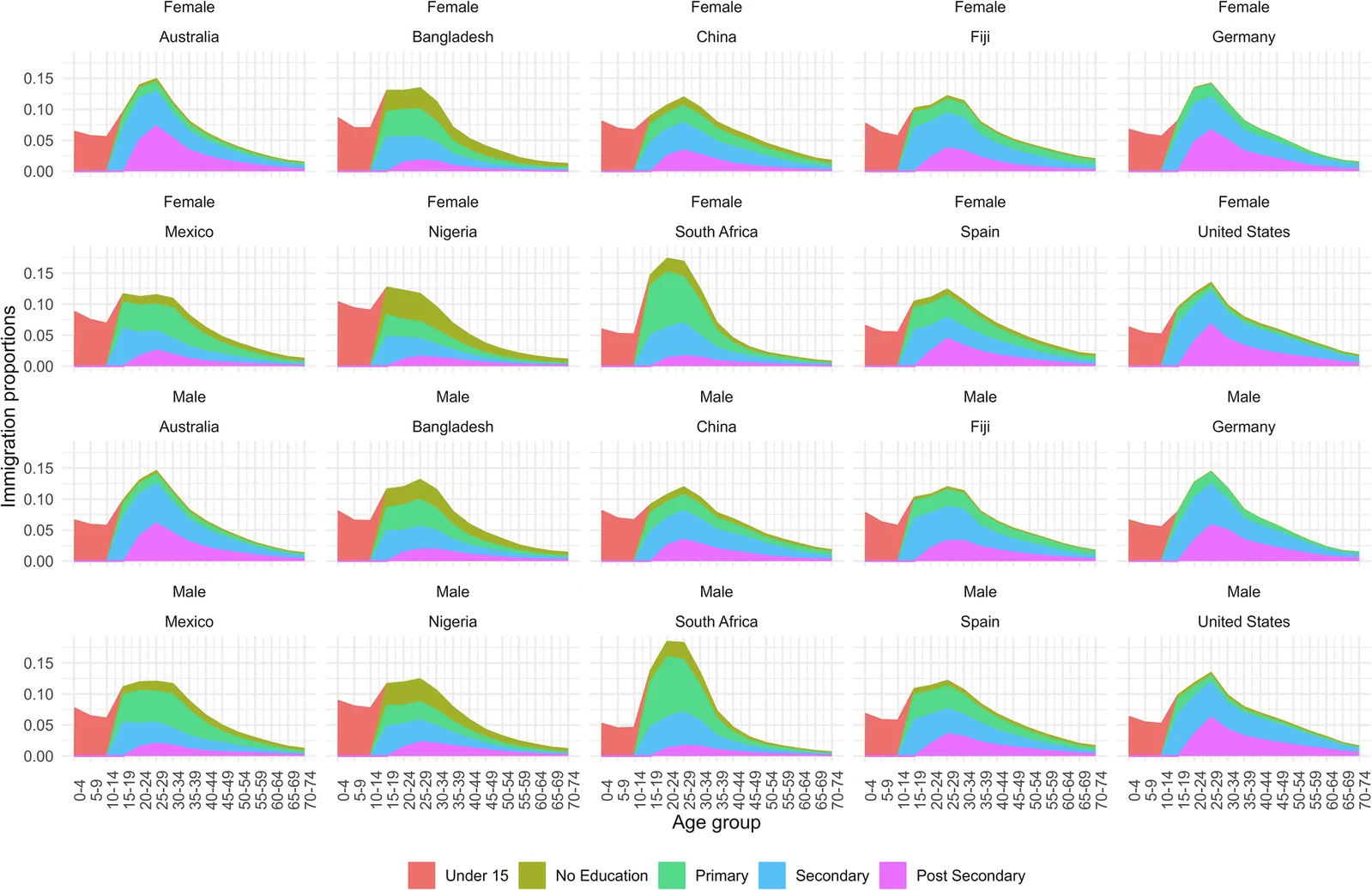
Predicted age and education composition of immigration flows in selected countries in 2015–2020.

Given the trained super learner model, we predicted the age-education proportions for male and female immigration flows for 199 countries during six five-year intervals from 1990–1995 to 2015–2020. Figure 3 illustrates our predicted age and education-specific compositions of female and male immigration flows for selected countries during the last five-year period. While the proportions of males and females by age and education are similar within a country, they differ significantly between countries. For instance, Australia and Germany have a high proportion of post-secondary educated immigration flows, whereas Bangladesh and Nigeria have a high proportion of flows with lower levels of education. Some countries, such as South Africa, have a higher proportion of young working-age immigration flows than the other selected countries. These results are not surprising, given the selective migration policies and the rise in the share of tertiary educated immigrants in OECD countries^57^. In contrast, the age and education composition of immigration flows to Bangladesh, Nigeria and South Africa are likely to be driven by the immigration from neighbouring countries^58^.

**Fig. 4:**
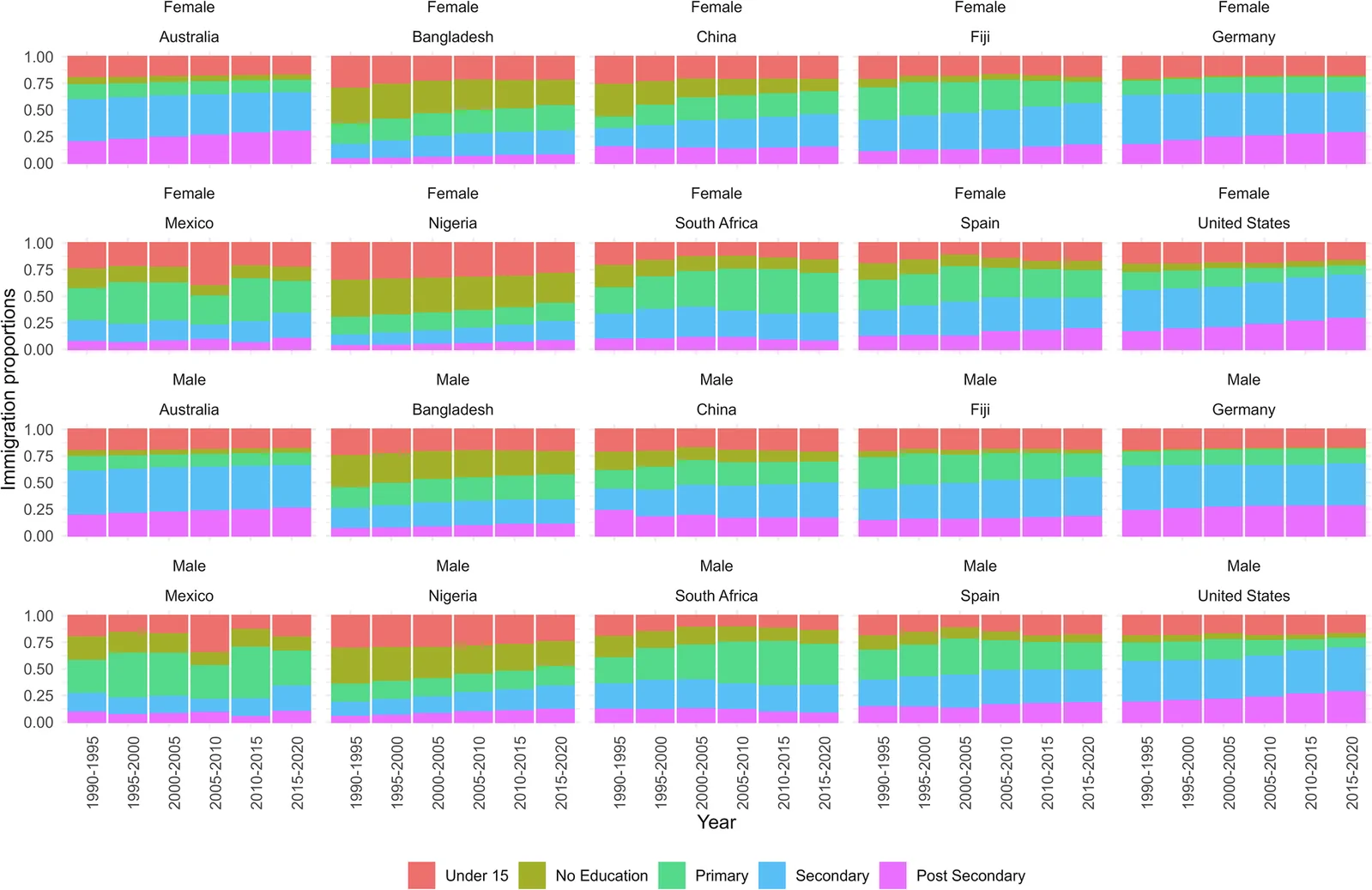
Predicted education composition of immigration flows in selected countries over time.

Figure 4 illustrates the proportion of female and male immigration flows by education level over time in selected countries. Again, there are no notable differences in the proportions of males and females within same country. However, there are differences between countries. For example, Australia, Germany and the United States have higher and increasing proportions of post-secondary education level, whereas China and Fiji have seen the greatest increase in the secondary education level. An interesting finding is the increase in Under 15 immigration flow in Mexico during 2005–2010, which also evident in the census samples and is possibly linked to the return migration from the United States^59^.

### Predictor variables

To produce a global dataset, we limit our selection of predictor variables to the indicators available from global databases providing information for a large number of countries over the last three decades. Population size and proportions by age, sex and education were collected from the Wittgenstein Centre Data Explorer^47^. The immigration stock and the size of diaspora are collected from the United Nations Migrant Stock 2020 data^60^. The GINI index was collected from GapMinder, which combines various sources to construct a global dataset^61^. Sex ratio, sex ratio at birth, sex specific life expectancies at birth and age 80, population density, median age, rate of natural change, population growth rate, births to 15–19 year old mothers, crude birth rate, total fertility rate, mean age at childbearing, crude death rate, infant mortality rate, under five mortality rate, net number of migrants, and net migration rate are extracted from the UN World Population Prospects 2024^29^. The Human Development Index, Gross National Income per Capita, adolescent birth rate, maternal mortality ratio, carbon dioxide emissions per capita (production), percentage of population with at least some secondary education, mean years of schooling, and expected years of schooling are collected from the United Nations Development Programme (UNDP)^62^. Missing cases for each explanatory variable were filled with the previous year’s value from the same country wherever possible, and the remaining empty cases were filled with the next year’s value.

One additional explanatory variable, related to the migration measurement, was included to reflect the varying migration intervals used to detect immigrants in the IPUMS census samples. As noted previously, in most censuses, these intervals were either one or five years, and they did not contribute greatly to predicted differentials in the age and education distributions.

**Fig. 5:**
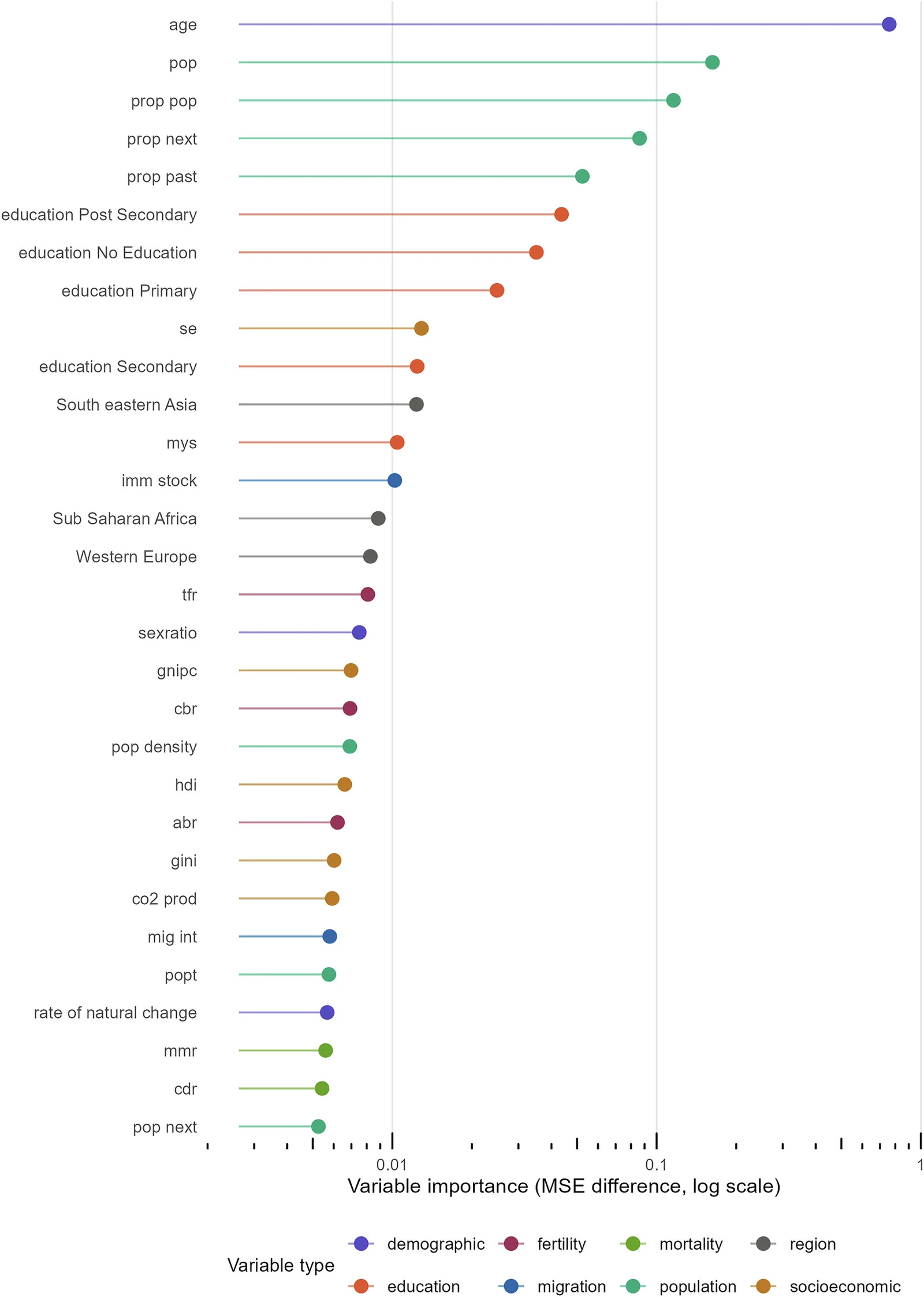
Variable importance.

In Fig. 5, we present the variable importance according to the MSE differences in the SL model. In this manuscript, the variable importance is reported solely for interpretation reasons and is not used for predictor selection. It is calculated using a permutation based approach instead of removing a variable, and shows how much the fitted SL model relies on the observed variable. The most important variable was calculated as age group followed by variables related to the population age and education structure.

## Data Records

Immigration flow compositions by age group, sex, and educational attainment and R codes are freely accessible on Zenodo^63^. The dataset *Immigration_composition_by_RCSL_20260520.csv* contains the results with separate rows for ISO 3 country codes (199 in total) for each combination of five-year period, sex (2 categories), age group (16 categories), and educational attainment level (4 categories) and immigration flow proportions (prop).

## Technical Validation

We validate our estimated migration flow proportions in four ways. First, we present the results of the cross-validation exercise. Second, we compare initial learner and the super learner algorithm using multiple evaluation metrics. Third, we compare our age composition estimates with equivalent measures supplied to Eurostat by European Free Trade Association (EFTA) countries from official migration statistics. Finally, we present Leave-One-Period-Out validation results.

Following the recommendations by Phillips *et al*.^64^, we first present the Cross-validated predictive performance (CV risk) for all initial learners alongside that of the super learner. The evaluation function was the squared loss error, which is equivalent to Mean Squared Error (MSE) averaged across all 10 folds. In Table 3, we compare our main model of applying RC migration schedules to the input data (SL with RC), as opposed to the super learner algorithm which utilised unprocessed input data (SL without RC). The super learner algorithm, which integrated RC migration age schedules, demonstrated superior performance compared to both the initial learners and the super learner algorithm that did not incorporate RC age schedules.

Second, we present in Table 4 multiple evaluation metrics to assess the performance of super learner algorithm and initial base learners including Root Mean Square Error (RMSE), Mean Absolute Error (MAE), Mean Absolute Percentage Error (MAPE) and the squared correlation between observed and predicted values (R2). For the autotuned RF the evaluation metrics are almost identical to the SL algorithm due to the large meta coefficient. For completeness, we also present in-sample MSE, calculated by comparing predicted values from the final SL model with the observed values. This metric is conceptually different from the cross-validated MSE (CV MSE) reported in Table 3, as it reflects model fit to the observed data rather than cross-validated generalization error.

**Table 4:** Evaluation metrics.

Model	MSE	RMSE	MAE	MAPE	R2
Ranger autotune	0,012	0,110	0,001	18,0	0,978
Ranger 5	0,037	0,192	0,002	38,3	0,934
Ranger 10	0,019	0,138	0,002	21,4	0,964
Ranger 15	0,016	0,126	0,002	19,1	0,970
Ranger 20	0,015	0,122	0,001	18,0	0,972
Ranger 25	0,014	0,118	0,001	18,0	0,972
Ranger 30	0,014	0,118	0,001	17,5	0,973
Ranger 35	0,014	0,118	0,001	17,3	0,973
Ranger 40	0,014	0,118	0,001	17,6	0,973
Ranger 45	0,014	0,118	0,001	17,4	0,972
Ranger nt1000	0,026	0,161	0,002	27,0	0,954
Ranger mn10	0,033	0,182	0,002	33,5	0,938
SuperLearner	0,012	0,110	0,001	17,9	0,978

Note: MSE and RMSE present 1000 x MSE and 1000 x RMSE.

**Fig. 6:**
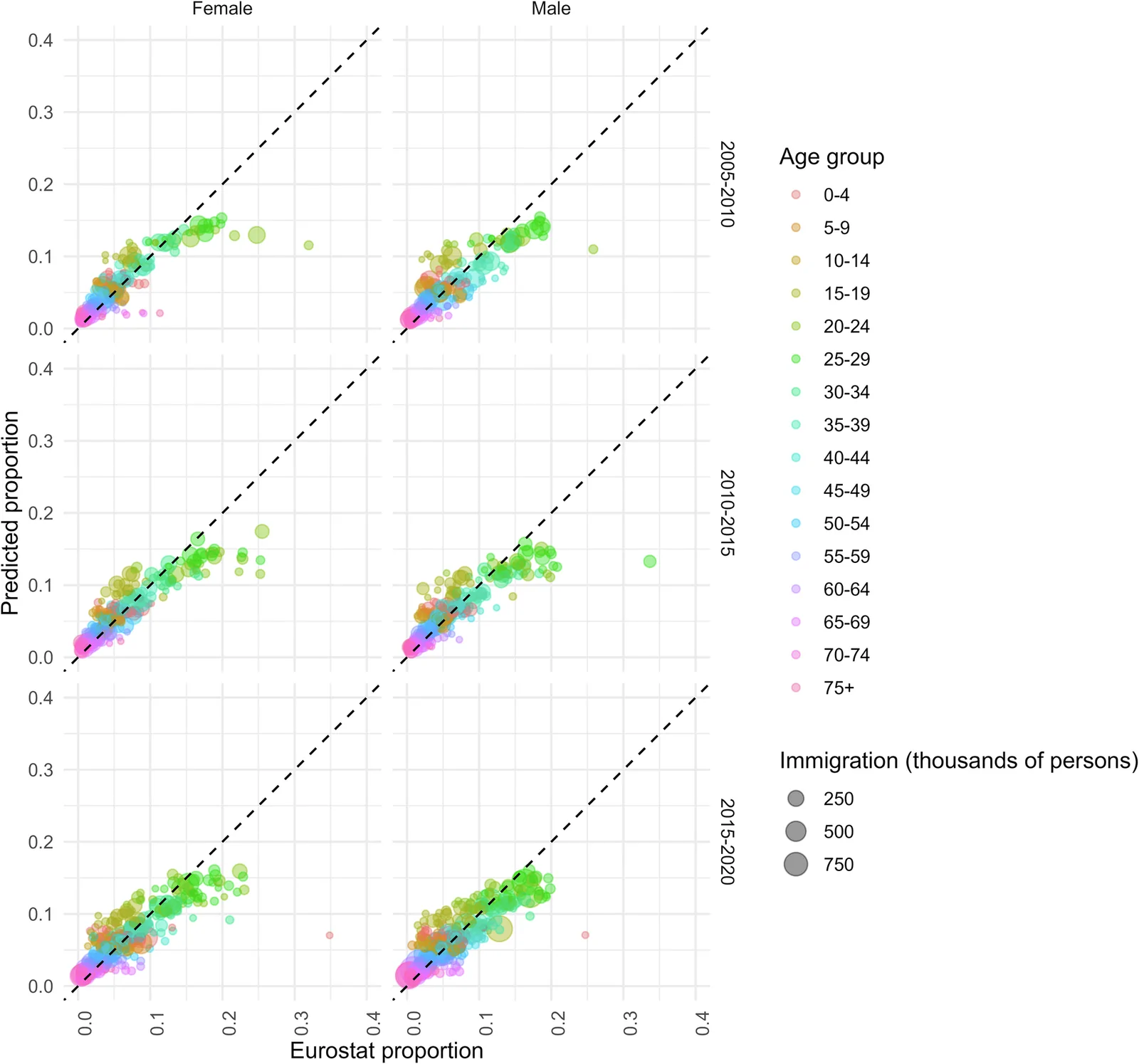
Comparison of predicted age composition of immigrants and five-year average Eurostat age composition for available EFTA countries in Eurostat. Note: 2005–2010: Denmark, Estonia, Germany, Netherlands, Norway, Slovakia, Sweden. 2010–2015: Belgium, Denmark, Estonia, Finland, Italy, Netherlands, Norway. 2015–2020: Belgium, Bulgaria, Croatia, Denmark, Estonia, Finland, Germany, Iceland, Italy, Lithuania, Netherlands, Norway, Slovakia, Spain, Sweden, Switzerland.

Eurostat publishes annual immigration flows by age and sex. Figure 6 compares five-year age-specific immigration proportions for the EFTA countries for 2005–2010, 2010–2015 and 2015–2020 periods calculated by averaging available Eurostat one-year proportions, with our estimates for the same five-year proportions. The most considerable differences between Eurostat and our estimates were found for the 0–4 age group immigrants in Slovakia (Eurostat = 0.35 vs SL = 0.07 for females, Eurostat = 0.25 vs SL = 0.07 for males), and 20–24 age group in Denmark (Eurostat = 0.32 vs SL = 0.12 for females, Eurostat = 0.26 vs SL = 0.11 for males). Such differences are important, as substantial deviations in a single age group can significantly affect the overall age composition of the population. The Pearson correlation coefficient between the five-year average Eurostat data (2005–2010, 2010–2015, 2015–2020) and the five-year estimated proportions for the same period is 0.86. Despite harmonisation efforts some differences remain in the immigration data reported to Eurostat by National Statistical Offices^5,65,66^. One such difference is related to the inclusion of live births occurring outside of the reporting country to the parents resident in reporting country. Czechia, the Netherlands, Poland and Slovakia include such births in the migration statistics whereas remaining countries do not. Another difference is that about half of the countries include asylum seekers and refugees in their migration statistics, while the remaining half exclude them. Such discrepancies inevitably affect the age composition of immigration flows especially in young age groups.

Finally, to assess SL model’s predictive capability and its ability to generalize to periods where training data is not available we use a Leave-One-Period-Out approach. For each five-year period, the SL was trained on the five of the six periods and the predicted proportions for the held out period were compared to the observed proportions. In Table 5, we present evaluation metrics. The model demonstrated consistent generalization across periods with average 0.0001 MSE.

**Table 5:** Evaluation metrics for the Leave-One-Period-Out validation.

Held out period	n	MSE x 1000	RMSE	MAE	R2
1990–1994	3072	0,090	0,009	0,005	0,701
1995–1999	2560	0,117	0,011	0,006	0,679
2000–2004	4800	0,121	0,011	0,005	0,661
2005–2009	3072	0,097	0,010	0,005	0,713
2010–2014	4224	0,103	0,010	0,005	0,695
2015–2019	384	0,105	0,010	0,005	0,761
Mean		0,106	0,010	0,005	0,702

Similar to RF models the main SL algorithm is strong in interpolation but limited in predicting values outside the range observed in the training data. In the training data, age-education-specific immigrant proportions rarely exceeded 0.20. Therefore, the model has limited exposure to high proportions. While the model is not entirely naïve to high values, predictions in high ranges should be interpreted cautiously.

## Supplementary information

Supplementary Information

## Data Availability

The results and the R codes to produce the estimates in this paper are available in Zenodo^63^. Due to data use agreements, the input data analysed in this study are not permitted to be shared publicly. IPUMS International data are accessible to researchers who register and obtain approval through the IPUMS International website. All predictor variables, prepared in 5_prep_gravity_imm.R file are available publicly and links to the datasets are provided both in this manuscript and in the R file.

## Code availability

The R codes to produce the estimates in this paper are available in Zenodo^63^. The R code includes 9 files: (1) To prepare the IPUMS International proportions, (2) To transform the IPUMS International proportions to immigration rates for the Rogers-Castro migration age schedule, (3) To implement the Rogers-Castro migration age schedule, (4) To transfer back the Rogers-Castro immigration rates to proportions, (5) to prepare the predictor variables, (6)To implement super learner algorithm, (7) To implement a more diverse super learner algorithm, (8) To implement a super learner algorithm to raw proportions before applying Rogers-Castro age schedule, (9) To create the maps.
